# Book Review

**DOI:** 10.1038/sj.bjc.6690612

**Published:** 1999-08


*British Journal of Cancer* (1999) **80**(11), 1864–1865
© 1999 Cancer Research Campaign
Article no. bjoc.1999.0612

### Book Review

### Cancer: How Worthwhile is Non-curative Treatment?

Edited by ML Slevin and T Tate

Oncology as a specialty appears to be growing exponentially, with establishment of specialist clinicians working in dedicated centres, armed with ever increasing options for treatment. Even so, little more than 10% of all adult cancers are currently cured and overall mortality rate has hardly changed over the last half century (Bailar and Gornik, 1997). Noteworthy, yet perhaps forgivable, is the fact that this dismal figure is rarely emphasized to the public. It is as painful to ourselves as to our patients to accept failure. Media headlines heralding early research findings as a 'cancer cure on the horizon' reflect the outcome we all wish to encounter in our lifetime, yet in reality, few such preclinical 'breakthroughs' achieve meaningful clinical benefit. Thus, most of the treatment administered for cancer will not cure our patients. We describe their treatment as 'palliative' in nature. But do we really know what form of palliation is achieved with treatment?

This book entitled, *Cancer: How worthwhile is non-curative treatment?*, edited by ML Slevin and T Tate, attempts to address this question. The editors make the point that considerable confusion and uncertainty surrounds the potential benefits and harms of treating patients with non-curable cancer. Thus, with the assistance of a series of specialist oncologists, a fairly stringent review has been conducted of the independent roles of surgery, radiotherapy and chemotherapy in palliating disease. It is no easy task, since, as many of the chapters independently discuss, high quality data in defining end points of palliation are hard to find.

These three key treatment modalities now occupy an unquestioned position in being considered to modify the natural behaviour of advanced cancer and so achieve effective palliation of disease. Yet, we need to address the question, just exactly what is achieved, and at what cost? The post-war experience when administering the first nitrogen mustards to lymphoma patients taught our predecessors important lessons which still plague us today: tumours regrow following visible tumour eradication and other solid tumours are far less chemo- and radio-sensitive. So this book has tried to identify what can be achieved by non-curative treatments in the context of the common cancers.

Derived from the Latin meaning 'cloak', 'to palliate' literally means to 'alleviate without curing'. Cancer specialists can classify cancers into those which are curable or incurable, and so offer our patients treatment with curative or palliative intent. The first problem the surgeons encounter is that most patients are operated on with 'curative intent', yet the statistical facts confirm that more often than not this is not achieved. The salient lesson from this observation is that surgeons need to discuss this difficult issue with their patients at the time of definitive surgery and then liase closely with their oncology colleagues, since multidisciplinary team working can benefit us all. The second problem is that few clinical trials of cancer surgery in the palliative setting have been undertaken. The third point is that while novel techniques such as lasers, cryoprobes and stents are now being introduced into clinical practice, evaluation in terms of symptom control is scarce.

The evidence of benefit with non-curative radiotherapy fares little better! The aims of palliative radiotherapy are varied. It may be implemented to achieve local control of asymptomatic disease with a view to improving quality of or prolong life. Alternatively, it may be used to alleviate symptoms. Well conducted, prospective and/or randomized trials to evaluate the former of these end points, are sadly lacking overall, despite the valiant efforts by Drs Sebag-Montefiore and Arnott to define the evidence-base in the context of, for example, gastrointestinal tract cancers. In contrast, the chapter describing radiotherapy for controlling symptomatic bone metastases is excellent instruction to the reader. The point is made that perhaps many patients too often think the aim of their treatment is prolongation of survival. These chapters should be read by oncologists who perhaps think likewise!

The aims of systemic therapy are equally variable: to delay or prevent tumour growth, prolong life, or relieve symptoms. Chemotherapy is, however, perhaps more often prescribed in hope than in expectation of benefit. Alison Jones' chapter on non-curative treatment of breast cancer honestly describes how chemotherapy became standard practice without undergoing clinical trials compared against best supportive care, yet only 40–60% of women will respond to first-line therapy and few patients will gain prolonged survival. What then is the value of this treatment? Can we define benefit in terms of quality of life as opposed to quantity of life? Alternatively, shouldn't we be working towards selecting patients more likely to respond? In the current climate, we also need to address the health economics of treatment and scrutinize carefully the validity of second and third line therapies in incurable patients. Is there a level of subtle coercion which causes doctors to administer probably futile chemotherapy at times when more appropriate support measures might be in the patient's interest? These rather provocative questions can only be properly answered if we know the true value of the palliative treatments being administered.

The chapter I recommend to you is Mike Cullen's account of how chemotherapy has been developed in the context of non-small-cell lung cancer (NSCLC). Clearly, most people would accept an amount of personal inconvenience and discomfort if the treatment intention is to cure, but what if a patient's life expectancy is around 12 months, despite treatment? Certainly, concerns regarding treatment-related toxicity, duration and practicality of administration have and still do result in failure of referral of cancer patients to specialist oncologists. It is therefore up to us to provide the evidence of what can be achieved by palliation of disease. In this chapter, Dr Cullen defines the goal of palliation in terms of the balance between quantity and quality of life. Quality of life must be considered in terms of practicalities of receiving treatment – hospital visits can be exhausting – and treatment-related side-effects must be weighed against the likelihood of controlling disease-related symptoms.

Since systemic therapy is probably the only modality likely to influence the natural history of NSCLC, it is right to pursue this line of approach. However, a meta-analysis of randomized trials of chemotherapy versus best supportive care has suggested marginal survival benefit of around 6 weeks. These data alone cannot justify chemotherapy as standard care. The data that clinch the argument in favour of treatment are comparison of patient symptomatology before and after a period of MIC chemotherapy. Dr Cullen describes the efforts to collect and analyse patient symptom data in the MIC trials, which was no easy task. Yet the data are priceless and the graphic presentation of symptom improvement with treatment needs no statistical number to justify itself. This chapter is a must for specialist registrars and consultants alike.

Overall, the style of this book is not optimal – segregation of treatment modalities normally considered together in a multidisciplinary approach makes reading rather cumbersome. The small font size and lack of illustrations makes it heavy reading for students, but the book is probably better dipped into than read cover-to-cover. Trainees are advised to bear in mind the speed of change in cancer management. For example, oral etoposide no longer has a place in treating poor-risk patients with SCLC.

Having read from cover-to-cover, this book leaves me with the distinct feeling of unease that oncology is practised predominantly as an art rather than a science in our country. Perhaps this is the best we can do at the current time, given our present evidence-base. As is emphasized many times in various chapters, there is an urgent need for more prospective studies to measure the benefit of non-curative treatment, both in terms of patient symptoms, toxicity and quality of life, alongside conventional end points of response and survival. This requires good quality clinical trials undertaken systematically by all practising oncologists – our service base must also be a data collection exercise if we are to properly define the role of the treatments we use on a daily basis. A recent clinical trial undertaken in the USA attempted to compare the role of palliative radiotherapy to stenting in advanced oesophageal cancer. The study closed with 276 patients entered during a period of time when at least 2700 new cases of this disease would have been diagnosed. This is simply unacceptable.

Finally, palliation of disease may constitute one or more of a number of goals. We must make it our duty both to clearly define these goals, and to know with some certainty the likely outcomes achieved with treatments offered to our patients. A requirement for education and constant reeducation must be our highest priority, since only in an atmosphere of knowledge can we achieve complete honesty in delivering the best care for our patients today and strive towards developing better treatments in the future.

### REFERENCE

Bailar JC and Gornik HL (1997) Cancer undefeated. *N Engl J Med* **336**: 1569–1574

*P de Takats*
*Department of Medical Oncology,*
*Addenbrooke's Hospital,*
*Hills Road, Cambridge CB2 2QQ*

### Letter to the Editor

### Quantification and prognostic relevance of angiogenic parameters in invasive cervical cancer

### Sir

We read with great interest the article by Tjalma et al (1998). The authors have investigated the angiogenic parameters in patients with cervical carcinoma whose menstrual states are not defined. The human endometrium undergoes a complex process of vascular and glandular proliferation, differentiation and regeneration with each menstrual cycle in preparation for implantation. Vascular endothelial growth factor (VEGF) is an endothelial cell-specific angiogenic protein that appears to play an important role in both physiological and pathological neovascularization (Goodger and Rogers, 1995; Shifren et al, 1996). As for the physiological neovascularization, there are two or three different endometrial angiogenic events during the human menstrual cycle, a post-menstrual repair, a mid–late proliferative growth and a lesser mid-secretory activity that may be associated with spiral arteriole growth (Rogers et al, 1992). The new capillaries formed in a malignant tumour are structurally similar to the capillaries growing during physiological neovascularization (Folkman and Klagsbrun, 1987). Patients with cervical carcinoma who are within the reproductive period of their lives, from menarche to menopause, the latter possessing wide variations in the age at which it occurs (Cunningham et al, 1993), still do menstruate, especially in the early stages. So a biopsy specimen obtained during, just before, or immediately after menstruation could be misleading. In this retrospective histopathological analysis, microvessel counts (MVC), which are representative of angiogenesis, are found to be either increased or decreased independent of menstruation. In conclusion, since the important impact of menstruation, physiological neovascularization, on MVC has not been mentioned it can be claimed that the results of this study must have been affected.

*İH Güllü and S Marangoz*
*Hacettepe University, Institute of Oncology,*
*Sihhiye, 06100, Ankara, Turkey*

### REFERENCES

Cunningham FG, MacDonald PC, Gant NF, Leveno KJ and Gilstrap LC III (1996) The endometrium and decidua: menstruation and pregnancy. In *Williams Obstetrics*, Cunningham FG et al (eds), pp. 81–109. Appleton and Lange: Connecticut

Folkman J and Klagsbrun M (1987) Angiogenic factors. *Science* **235**: 442–447

Goodger AM and Rogers PA (1995) Blood vessel growth in the endometrium. *Microcirculation* **2**: 329–343

Rogers PA, Abberton KM and Susil B (1992) Endothelial cell migratory signal produced by human endometrium during the menstrual cycle. *Hum Reprod* **7**: 1061–1066

Shifren JL, Tseng JF, Zaloudek CJ, Ryan IP, Meng YG, Ferrara N, Jaffe RB and Taylor RN (1996) Ovarian steroid regulation of vascular endothelial growth factor in the human endometrium: implications for angiogenesis during the menstrual cycle and in the pathogenesis of endometriosis. *J Clin Endocrinol Metab* **81**: 3112–3118

Tjalma W, Van Marck E, Weyler J, Dirix L, Van Daele A, Goovaerts G, Albertyn G and van Dam P (1998) Quantification and prognostic relevance of angiogenic parameters in invasive cervical cancer. *Br J Cancer* **78**: 170–174

© 1999 Cancer Research Campaign
